# Significant efficacy and well safety of apatinib combined with radiotherapy in NSCLC: Case report

**DOI:** 10.1097/MD.0000000000009276

**Published:** 2017-12-15

**Authors:** Chunbo Zhao, Qian Zhang, Wenbo Qiao

**Affiliations:** Department of Radiation Oncology, The Third Affiliated Hospital of Harbin Medical University, Harbin, Heilongjiang Province, China.

**Keywords:** apatinib, case report, lung cancer, thoracic radiotherapy

## Abstract

**Rationale:**

The outcomes of locally advanced non-small cell lung cancer (NSCLC) remain poor, in particular, the frail elderly patients cannot tolerate chemotherapy. The new efficient, safe, and more specific treatments are needed. Radiation combined with targeted therapy is the focus of research in recent years. Apatinib is highly selective tyrosine kinase inhibitor of vascular endothelial growth factor receptor-2, studies have revealed that apatinib inhibit the growth of solid tumors including NSCLC. However, there is no report to evaluate its efficacy and safety in combined with radiotherapy for the advanced NSCLC. Our original research about to explore the use of apatinib combined with radiotherapy in treatment of NSCLC and its side effects are as follows.

**Patient concerns:**

Patient 1, man, 78-year old, admitted to hospital, due to “thoracalgia and dyspnea for 1 month.” Chest and abdomen computed tomography (CT) scan showed that there was a huge mass at the left upper lobe and multiple lymph nodes metastasis in mediastinum and left hilus pulmonis, the diagnosis was left lung squamous cell carcinoma, however, the mass was huge and age of patient was elder, post chemotherapy the mass were bigger and more severe. Patient 2, man, 61-year old, the diagnosis was squamous carcinoma on left upper lobe with right mediastinum lymph notes metastases recrudescence post chemoradiotherapy.

**Diagnoses:**

Case 1 was diagnosed left lung huge squamous cell carcinoma and case 2 was left lung squamous carcinoma, the primary lesion and right mediastinum lymph notes metastases recrudescence after radiochemotherapy.

**Interventions:**

Both patients who received local radiation therapy and concurrent apatinib. Apatinib 250 mg once daily in combination with thoracic radiotherapy (2 Gy/d, 5 fractions/wk) followed by Apatinib Maintenance Therapy.

**Outcomes:**

Favorable oncologic outcomes were achieved in the 2 cases after the treatment. The common side effects of apatinib were hypertension and hand-foot syndrome; however, the toxicity of was controllable and tolerable, no dyspnea, no hemoptysis, no thoracalgia.

**Lessons:**

Apatinib combined with thoracic radiotherapy, may be an option for recurring or advanced NSCLC. But that still warrants further investigation in the prospective study.

## 1 Introduction

The standard treatment for locally advanced unresectable non-small cell lung cancer (NSCLC) is the association of conventional chemotherapy (platinum based doublets) and radiotherapy.^[1,2]^ Outcomes of locally advanced NSCLC, however, remain poor and new efficient, safe, and more specific treatments are needed. As we know, angiogenesis is a key process for cell growth, especially for the tumor growth.^[3]^ And the vascular epidermal growth factor (VEGF) can activate the downstream pathway to stimulate the proliferation of vessel endothelium via binding vascular epidermal growth factor receptor (VEGFR), thus leading to the growth of tumor. Studies have revealed that antiangiogenesis drugs inhibit the growth of solid tumors including NSCLC.^[4]^

As the first generation of oral antiangiogenesis drug created in China, apatinib which targets mainly at VEGFR-2 has a significant effect on the treatment of the advanced gastric carcinoma, significantly prolonging overall survival time (OS) of the advanced gastric cancer patients who failed in the second-line treatment. Apatinib has been known for its simplicity, compliance, and less side effects. Recently, more and more clinical practices are using apatinib in advanced metastatic gastric cancer and breast cancer. However, there is no report to evaluate its efficacy and safety in combined with radiotherapy for the advanced NSCLC. Herein, the cases for the advanced NSCLC using Apatinib concurrent local radiation therapy for advanced NSCLC in our hospital are as follows.

## 2 Cases presentation

**Figure 1:**
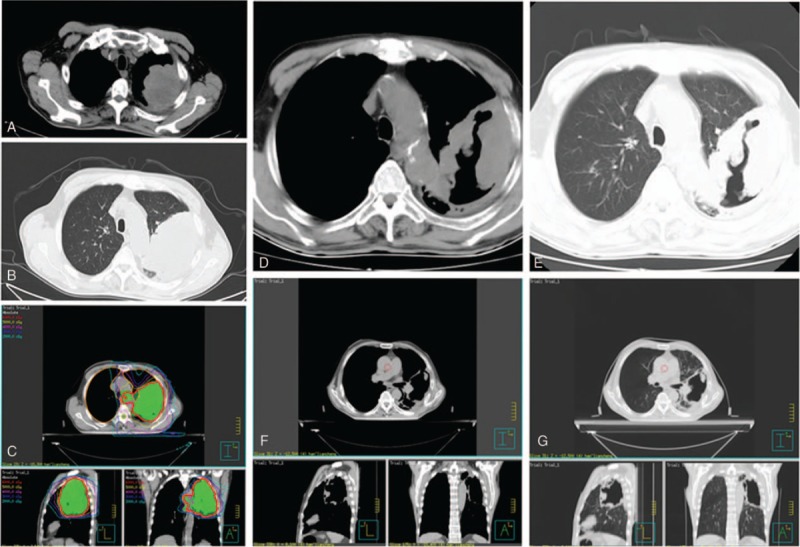
A and B: CT scan showed that there was a huge mass at the left upper lobe multiple lymph nodes metastasis in mediastinum and left hilus pulmonis. C: The target range of irradiation including the left lung primary tumor and mediastinal lymph node metastasis (V-MAT 2 Gy/F total DT: 60 Gy/30F). D and E: 1 month after treatment (January, 2017) CT scan showed that therapeutic evaluation was partial remission (PR) and the mass reduced partially. F and G: Three months after treatment (April, 2017) CT scan showed that the interior of the tumor was necrosis, cavity formation, wall nodules formation. CT = computed tomography.

Patient 1, man, 78-year old, admitted to hospital on October 15, 2016, due to “thoracalgia and dyspnea for 1 month.” Chest and abdomen computed tomography (CT) scan showed that there was a huge mass at the left upper lobe and multiple lymph nodes metastasis in mediastinum and left hilus pulmonis, puncture biopsy guided by CT result was “squamous cell carcinoma on left upper lobe” (Fig. 1A and B). Cranium MRI indicated that there was no space-occupying lesion. Abdomen were not found metastatic lesion. No gene mutations were detected in Anaplastic Lymphoma Kinase (ALK) or Epidermal Growth Factor Receptor (EGFR) examinations. The diagnosis was left lung squamous cell carcinoma with no metastases, however, the mass was huge and age of patient was elder, which was treated by chemotherapy of single docetaxel for 1 cycle (October 20, 2016). CT scan (November 13, 2016) indicated that compared with the previously one, the mass at the left upper lobe were bigger and more severe. The therapeutic evaluation was progressive disease (PD) (RECIST version 1.0). The patient started oral administration of apatinib (250 mg/d) concurrent local radiotherapy (V-MAT 2 Gy/fraction total DT: 60 Gy/30 fraction); post-radiotherapy Apatinib Maintenance Therapy (Fig. 1C). One month after treatment (January 27, 2017), CT scan showed that therapeutic evaluation was partial remission (PR), and the mass reduced partially (Fig. 1D and E). Three months later, CT scan showed that therapeutic evaluation PR and the mass further reduced partially. The interior of the tumor was necrosis, cavity formation, wall nodules formation (Fig. 1F and G). Apatinib Maintenance Therapy, this case's progression-free-survival (PFS) is keep on follow up, the patient no dyspnea, no hemoptysis, no thoracalgia.

**Figure 2:**
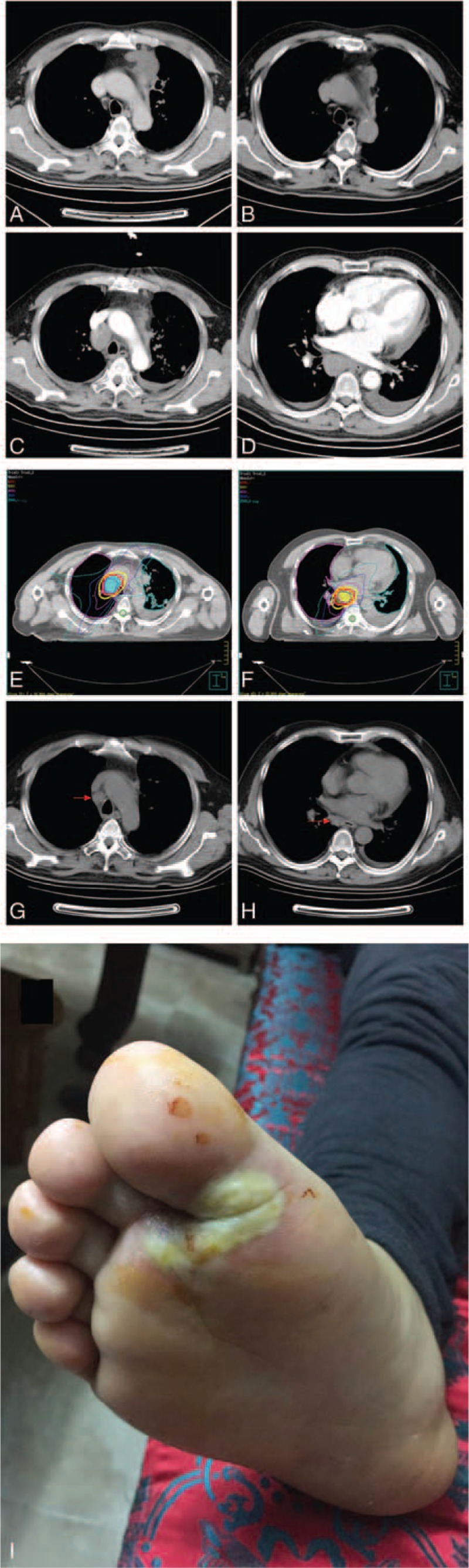
A: CT scan showed that there was a mass on left upper lobe (September 10, 2015); B: follow up CT scan showed that the mass on left upper lobe reduction; C and D: CT scan showed that there was lymph nodes metastasis in mediastinum (3A prevascular and right pulmonary ligament). Pleural effusion occurred at the thoracic cavity. Left upper lobe residual mass was steady; E and F: Radiotherapy (IMRT 2 Gy/fraction total DT: 40 Gy/20 fraction); G and H: One months later, CT scan showed that metastases lymph notes of mediastinum (3A prevascular and right pulmonary ligament) reduced partially, therapeutic evaluation partial remission (PR) and the mass of Left upper lobe residual reduced partially as well and pleural effusion reduced; I: The common side effects of apatinib was hand-foot syndrome. CT = computed tomography.

Patient 2, man, 61-year old, was examined by broncho-fiberscope (September 10, 2015) in local hospital due to “Irritating dry cough for more than one month.” Pathological report revealed that it was squamous carcinoma on left upper lobe (Fig. 2A). No gene mutations were detected by EGFR examinations. Cranium MR indicated that there was no space-occupying lesion. Abdomen were not found metastatic lesion. With chemotherapy of “docetaxel and cisplatin” for 2 cycles (September 20, 2015 to November 4, 2015), the therapeutic evaluation was PR. It was sequential local radiotherapy for left lung upper mass (IMRT 2 Gy/fraction total DT: 60 Gy/30 fraction). Afterward Chinese medicine was used for treatment, follow up CT scan shows the mass reduction (Fig. 2B). One year later (December 10, 2016), admitted to hospital for check the cancer, chest and abdomen CT scan showed that there was lymph nodes metastasis in mediastinum (3A prevascular and right pulmonary ligament) and pleural effusion occurred at the thoracic cavity, left upper lobe residual mass was steady (Fig. 2C and D), abdomen were not found metastatic lesion. The result of bone ECT was normal. The diagnosis was left lung squamous cell carcinoma with right mediastinum lymph notes metastases recrudescence. The patient started oral administration of apatinib (250 mg/d) concurrent local radiotherapy (IMRT 2 Gy/fraction), concurrent took apatinib orally (250 mg/d) (December 20, 2016) (Fig. 2E and F). When patient accepted total dose 40 Gy/20 fraction, occurred cough, slight dyspnea. Worry about radiation pneumonitis occurring, end radiotherapy, post-radiotherapy Apatinib Maintenance Therapy. One month after treatment (February 22, 2017), CT scan showed that metastases lymph notes of mediastinum (3A prevascular and right pulmonary ligament) reduced partially, therapeutic evaluation PR and the mass of Left upper lobe residual reduced partially as well, and pleural effusion reduced (Fig. 2G and H). Follow up Apatinib Maintenance Therapy, the patient no dyspnea, no hemoptysis, no thoracalgia, but hand-foot syndrome (Fig. 2I). However, the toxicity of was controllable and tolerable.

## 3 Discussion

Studies revealed that new vessels provide nutrient and oxygen for tumors. New blood vessel formation or neovascularization is crucial for tumor growth and metastasis. By restraining angiogenesis, it is possible to inhibit the growth of tumor, development, and metastasis. VEGF/VEGFR is an important set of ligand and receptor affecting the angiogenesis, which is over expressed on the surface of various tumors. In recent years, researchers have been trying to restrict the growth of tumor by restraining the combination between VEGF and VEGFR to stop the activation of the downstream pathway.^[5]^ The targeted therapy of antiVEGF/VEGFR includes reducing the concentration of activated and freed VEGF and cutting off the VEGFR signal system.

Traditionally, the standard regime for advanced NSCLC is chemotherapy of 2 drugs based on platinum and palliative radiation therapy. It brings up a point that chemo-radiotherapy has reached the plateau period.^[6]^ At the end of the 20th century, the status of angiogenesis in lung cancer has been confirmed by several studies. Some studies found out that the increase in the density of capillaries is closely related to the growth of tumor and poor prognosis.^[7,8]^ The emergence of antiangiogenesis drugs brings hope to patients with advanced NSCLC.

Apatinib is the first generation of oral antiangiogenesis drug invented in China, Apatinib targets the intracellular ATP-binding site of VEGFR-2, as well as the receptor tyrosine kinase (RTK) such as c-kit, RET, and c-src.^[9]^ It was found that VEGF-2 promoted endothelial proliferation by activating the mitogen-activated protein kinase (MAPK) signaling pathway during the process of angiogenesis.^[10]^ And by blocking VEGFR-2, apatinib can suppress endothelial proliferation and finally lead to antiangiogenesis, which was confirmed to exerted an antitumor effect on various cancers.^[11]^

A study has revealed that apatinib showed significant competence in treating solid tumor. In the study, 45 patients with measurable tumors originated from lungs, gastrointestinal tract, and other organs were randomly assigned to the oral apatinib groups of 250, 500, 750, 800, and 1000 mg/d. After taking in apatinib, 7 patients achieved PR (18.9%) and 24 were with SD (64.9%).^[12,13]^ Upon the approval by China Food And Drug Administration, apatinib has been suggested to treat advanced gastric adenocarcinoma and adenocarcinoma in the gastroesophageal junction in China. To explore the further use of apatinib, a phase II, multicenter, placebo-controlled trial recruited 135 advanced nonsquamous NSCLC patients who failed over 2 lines of treatment. The patients received apatinib (750 mg/d) or placebo with allocation ratio of 2:1. The end point was the disease progression or unacceptable toxicity. The results showed that median PFS, response rate, and disease control rate in the apatinib group were all better than the placebo group (4.7 months vs 1.9 months, *P* < .0001; 12.2% vs 0%, *P* < .0158; 68.9% vs 24.4%, *P* < 0.0001, respectively). The most frequent adverse effects were hypertension, proteinuria, and hand-foot syndrome, all of which were manageable.^[14]^ Zhu and Li have reported that Apatinib, a new small molecular VEGFR2 inhibitor suppresses the activity of lung cancer stem cells in the 2016 WCLC meeting, provide an important molecular basis for the application of Apatinib in lung cancer patients.^[15]^

The reasons for the use of apatinib combined with local radiotherapy in our present study were due to intolerant side effects of chemo-radiotherapy or unsatisfactory treatment efficacy and second radiotherapy for recurrent lung cancer, but patients still have strong wish to continue treatment. According to the studies have revealed that anti-angiogenic drugs can make tumor blood vessels normalization, remission of tumor hypoxia, down regulation of vascular endothelial growth factor, and combined induction of tumor cell apoptosis.^[16]^ We treated them with apatinib in daily dose of 250 mg and combined with local radiotherapy. After a period of treatment, the disease was well controlled for a certain time. Although the 2 cases mentioned above were individual, apatinib did show its curative effect combined with radiotherapy in NSCL cancer. In our cases, the common side effects of apatinib were hypertension and hand-foot syndrome; however, the toxicity of apatinib was controllable and tolerable, hand-foot syndrome with grade 2 occurred and it was well managed. What is noteworthy is that the 2 patients were squamous carcinoma benefited from apatinib, then remains to be determined. Previous clinical trials assessing Apatinib for advanced NSCLC did not include with irradiation. There is a need for more data to create treatment guidelines that can be refined to maximize treatment benefit and minimize toxicity.

## 4 Conclusion

Apatinib did show its curative effect combined with radiotherapy in NSCL cancer. The common side effects were hypertension and hand-foot syndrome; however, the toxicity was controllable and tolerable. There is need of more clinical trials to assessing Apatinib combined with radiotherapy for NSCLC to maximize treatment benefit and minimize toxicity.
